# A Comparison of the Nutritional Qualities of Supermarket’s Own and Regular Brands of Bread in Sweden

**DOI:** 10.3390/nu12041162

**Published:** 2020-04-22

**Authors:** Veli-Matti Lappi, Antoine Mottas, Johan Sundström, Bruce Neal, Marie Löf, Karin Rådholm

**Affiliations:** 1 Department of Biosciences and Nutrition, Karolinska Institutet, SE-141 83 Stockholm, Sweden; vmlappi.research@gmail.com (V.-M.L.); antoine.mottas72@gmail.com (A.M.); marie.lof@liu.se (M.L.); 2 Department of Medical Sciences, Uppsala University, SE-741 85 Uppsala, Sweden; johan.sundstrom@medsci.uu.se; 3 The George Institute for Global Health, University of New South Wales, Sydney 2042, Australia; bneal@georgeinstitute.org.au; 4 Department of Epidemiology and Biostatistics, Imperial College London, London SW7 2AZ, UK; 5 Department of Health, Medicine and Caring Sciences, Linköping University, SE-581 83 Linköping, Sweden

**Keywords:** health star rating, FoodSwitch, supermarket’s own brands, private label, gluten-free, front-of-pack labelling, bread, nutritional quality, packaged food

## Abstract

Processed food is associated with unhealthy qualities such as higher content of harmful fats, sugars and salt. The aim of our study was to compare the nutritional qualities of supermarket’s own brands and regular brands of bread sold in Sweden. Additionally, we compared the nutritional qualities of gluten-free and gluten-containing bread. We collected information from the labels of 332 bread products available in the largest grocery store chains. The Australian Health Star Rating (HSR) system was used to quantify the nutritional quality of each bread product. We compared all supermarket’s own brand products to regular brand products, and gluten-free to gluten-containing bread. The mean HSR for the supermarket’s own brands was lower than the regular brands (3.6 vs. 3.7; *p* = 0.046). For the regular brand products, the fibre, sugar and total fat content were greater (*p* < 0.001, *p* = 0.002 and *p* = 0.021, respectively), while less protein (*p* = 0.009) compared to regular bread products. Gluten-free bread had a lower HSR than gluten-containing bread (mean 3.5 vs. 3.8, respectively; *p* < 0.001). The regular brand products were slightly healthier than the supermarket’s own brands, primarily as a result of a higher fibre content. Gluten-free bread products were slightly unhealthier due to a lower protein content.

## 1. Introduction

In 2017, dietary risk factors attributed to at least 19% of all global deaths [1,2,3]. A poor diet has been linked to non-communicable diseases including obesity, coronary heart disease, cancer and diabetes [1,4]. Obesity is on the rise worldwide and, therefore, means to assist in changing unhealthy dietary habits are urgently needed [5,6]. Obesity and the consumption of snack-like, processed and convenient food products have increased in many high-income parts of the world, and are now growing rapidly in low- and middle-income countries [7,8,9,10,11]. A study from New Zealand showed that the ultra-processed food represents the majority of packaged food products on the market [12]. There is an inverse association between level of processing and the healthiness of the nutrient profile [11,12]. Moreover, processed food tends to contain higher amounts of harmful fat, sugar and salt and less of fibre [11,13], which have been linked to adverse health outcomes [4,14,15]. On the other hand, commonly consumed bread, is often processed and packaged but depending on its contents it can be considered as part of a healthy diet [14,16].

In 2011, the European Union (EU) published a regulation which dictates how the nutrition labelling on packaged food items should be displayed [17]. The mandatory nutrition information panel (NIP) in the EU includes the amounts of energy, fat, saturated fat, carbohydrate, sugars, protein and salt [17]. In addition to the NIP, front-of-the-pack labelling is another way to inform consumers of the healthiness as well as environmental sustainability of products. Thus, the World Health Organisation has issued a European Food and Nutrition Action Plan 2015–2020 [18] in which they suggest easy-to-understand labelling as a tool to reduce the burden of diet-related diseases in Europe [18]. 

Until 2018, fifteen EU countries had voluntarily adopted a policy considering front-of-pack labelling [19]. Thirteen of these countries had various front-of-pack endorsement logos to help consumers choose healthier products [19]. In Sweden, the Swedish National Food Agency has registered the Keyhole symbol as a trademark which acts as a sign of healthier foods [20]. This symbol is based on the Nordic Nutrition Recommendations [14] and has been introduced in Denmark, Norway and Iceland since it was first used in Sweden in 1989. It is likely that the Keyhole symbol has guided consumers towards healthier food choices during the years [7].

In Australia, a publicly funded Health Star Rating (HSR) system designed for labelling was developed in 2014 [21]. The purpose of the HSR system is to rank (from 0.5 to 5 stars with half star increments) packaged food products according to their healthiness. HSR is modified from Nutrient Profiling Scoring Criterion developed by governmental institutions of Australia and New Zealand [22,23]. HSR uses an algorithm that takes into consideration the negative aspects of energy, saturated fat, added salt (or sodium) and added sugar; while also accounting for the positive aspects of dietary fibre, protein, fruits, nuts, legumes and vegetable content [24]. In the HSR system, each packaged food item is categorized in one of six categories depending on type of food. Packaged bread products are all assigned to the same category (Supplementary Table S1). The HSR score is calculated according to the following equation: HSR score = Baseline points - Modifying points. Baseline points depend on energy content, saturated fat, sugar and salt and modifying points are based on protein, fibre, fruit and vegetable content (Supplementary Table S2-S3). The final assignment of HSR score depend on which category the product is assigned to (Supplementary Table S4) [24]. If product package does not have information vital to the algorithm, it uses an average of that nutrient belonging to the same category of foods in the database. The HSR has since been shown to align broadly with the Australian dietary guidelines [25,26] and it has been increasingly adopted and showed to have a benefit on consumer understanding about the healthiness of foods, though clear effects on consumer purchases in supermarkets remain to be proved [27,28]. Nutrient profiling (e.g., HSR) may also have the potential to influence the food industry to reformulate their products to healthier ones [29,30]. 

The FoodSwitch platform, including the mobile application FoodSwitch, enables consumers to assess the HSR of different packaged products [31]. It was developed to guide consumers to purchase healthier products. To date, it has been introduced to nine countries: Australia, China, Fiji, Hong Kong, India, New Zealand, South Africa, United Kingdom and USA [31]. The application is developed by the George Institute for Global Health and was released in 2014. With the application, consumers are able to scan the barcode of any packaged food product and get nutritional information for the product as well as a suggestion of healthier options derived from the regularly updated database. Data are collected by employees on a regular basis and through crowdsourcing; consumers voluntarily submitting data on products that are missing [32,33]. The multinational FoodSwitch database to date holds more than 700,000 products. 

Supermarket’s own brands are brands that belong to the retailers or distributors and are sold in their own stores [34,35]. They differentiate from regular brands which are owned by the original manufacturer or company and sold to supermarkets [34]. The supermarket’s own brand products can be manufactured by the retailer itself or ordered from another manufacturer to be sold as an own brand [34]. In Sweden, the majority of the grocery store market is covered by three largest retailer chains: ICA, Axfood and COOP [36] and all of these chains have several of their own brands. In addition, having the 5th largest market share in Sweden, LIDL is known to offer most of its products as their own brands [37]. As the market share of supermarket’s own brands increases and as much as 76% of EU citizens consider them to be good value for the money, it is important to assess the quality of these products [34,35,38]. 

Similarly to supermarket’s own brands, gluten-free products are becoming more available and the trend is increasing [39]. Additionally, consumers often perceive gluten-free food as a healthier option [40]. However, concerns regarding the health aspects and nutritional qualities of gluten-free products have emerged in the scientific community [41]. Gluten-free products generally have a higher fat and saturated fat content, while lower in protein compared to the equivalent gluten-containing products. A higher sugar content in gluten-free products compared to gluten-containing products have been suggested, but this is not a consistent finding [41].

Our aim was to investigate the healthiness (i.e., HSR) and nutritional qualities of the supermarket’s own brand packaged bread products in Sweden compared to regular brands. A secondary aim was to evaluate if there are significant differences between gluten-free and gluten-containing bread products.

## 2. Materials and Methods

This was a cross-sectional study conducted in Stockholm, Sweden. Data on all packaged bread products available for purchase were collected in January and February 2019 in four large supermarkets (ICA, Hemköp, COOP and LIDL).

The on-pack bar code was scanned and the nutritional information photographed and inserted into the database by two data collectors (V.L. and A.M.). If the on-pack information was unclear, a double check against the manufacturer’s website was done. All baked bread or ready-made mixed flours designed for baking a bread were included. Dry and hard bread were excluded. In detail, the categories included were soft bread (all shapes, sizes and contents), tortillas, pizza bases, ready-made flour mixes, bagels, soft taco shells, pita bread and filled garlic bread. 

Data were collected using a Data Collector Application (DCA, version 1.6) downloaded to Samsung Galaxy S8 mobile phone (Android system) developed by The George Institute for Global Health in Sydney, New South Wales, Australia. This specific DCA was designed to scan barcodes, take photos and submit data to the Content Management System (CMS) database. The CMS database was administered by The George Institute for Global Health. The CMS acts as the data source for FoodSwitch platform. Permission to collect data was requested from the store manager (if available) or the employees.

The information (barcode, NIP, ingredients and possible claims; gluten status and Keyhole symbol) from the products were entered manually in the CMS database. To minimize possible entering errors, one researcher entered the information (V.L.) and it was then double-checked (by A.M). The products were categorised according to a predetermined category structure in the CMS database. 

Supermarket’s brands were searched from the chains’ websites [42,43,44,45] or further inquired via email and then grouped accordingly into 2 categories: supermarket’s own brands and regular brands. A Keyhole assessment was done to see its relation to HSR. For categorisation purposes and comparison with the Keyhole branding, the products were dichotomized to healthy and unhealthy according to their HSR. In alignment with previous research, an HSR of ≥3.5 was considered as healthy and HSR of <3.5 as unhealthy [25,46]. If the NIP displayed only sodium content it was converted to corresponding amount of salt using a converter [47]. 

Kolmogorov–Smirnov (and Shapiro–Wilk) tests were performed to evaluate whether data were normally distributed. In addition, distribution was checked visually from histograms. Medians with corresponding IQRs and means with standard deviations (SD) were calculated. Differences between groups were evaluated using a Mann–Whitney U-test for skewed data and a Chi-Square test (Pearson Chi-Square or Fisher’s exact accordingly) for categorical variables. A value for significance was set to *p* < 0.05. The SPSS (IBM SPSS Statistics for Windows, Version 25.0) program was used to analyse the data.

## 3. Results

In total, we collected information from 332 different bread products from four major Swedish supermarkets which together represent over 90% of the total market [36]. One (Willy’s) out of five visited stores refused to give us access to examine their products. One product had an unreadable barcode which could not be uploaded to the database. The fibre content was not displayed in the NIP in 35 (11%) products, where 12 were supermarket’s own brand and 23 were regular brand products (*p* = 0.43). The Keyhole symbol was found on 43 (13%) products and 83 (25%) products were declared gluten-free. According to the HSR cut point of 3.5 stars, 292 (88%) were categorised as healthy and 40 (12%) as unhealthy. 

We found that 29% of the total bread products were supermarket’s own brand products comprising 11 different brands. The supermarket’s own brands had significantly higher protein, lower total fat, lower sugar and lower fibre content than the regular brands (Table 1). There was a significantly lower HSR for the supermarket’s own brands than for the regular brands. The sensitivity analysis that excluded products not displaying fibre content on the NIP showed the same result; a lower HSR for the supermarket’s own brands than for the regular brands. The proportion of both healthy and unhealthy products did not significantly differ between supermarket’s own brands and regular brands (*p* = 0.56).

**Figure 1:**
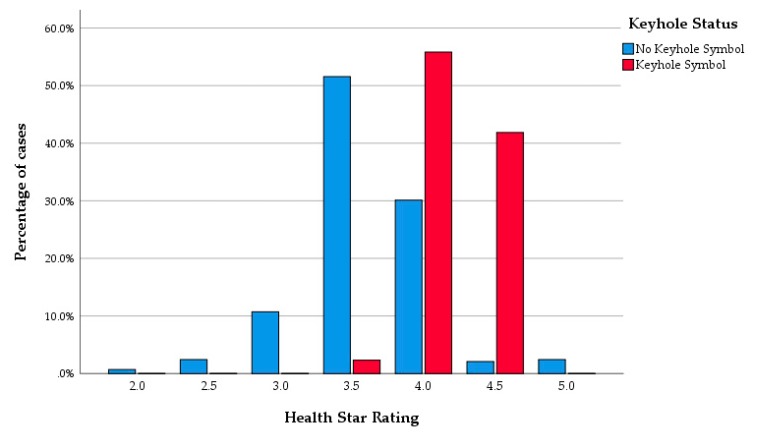
Health Star Rating of products according to Keyhole status.

The Keyhole status and HSR health categorisation were aligned, with all 43 Keyhole branded products receiving an HSR of 3.5 or more. The Keyhole products had significantly higher HSR than the products lacking the symbol (*p* < 0.001; with a mean HSR of 4.2 and 3.6, respectively) shown in Figure 1. The Keyhole marked products had a significantly lower energy density, less carbohydrates, less sugar and more fibre than non-Keyhole products. The amount of protein, total fat, saturated fat and salt did not significantly differ between products that were Keyhole marked or products not Keyhole marked (data not shown). All the Keyhole marked products were classified as healthy by the HSR. The proportion of Keyhole products did not significantly differ between supermarket’s own brands and regular brands (*p* = 0.12). The Keyhole symbol was on 43 (13% of total) products and 42 of these had HSR value of 4.0 or above. In addition, there were 100 more products without the Keyhole symbol that scored HSR of 4.0 or above.

**Figure 2:**
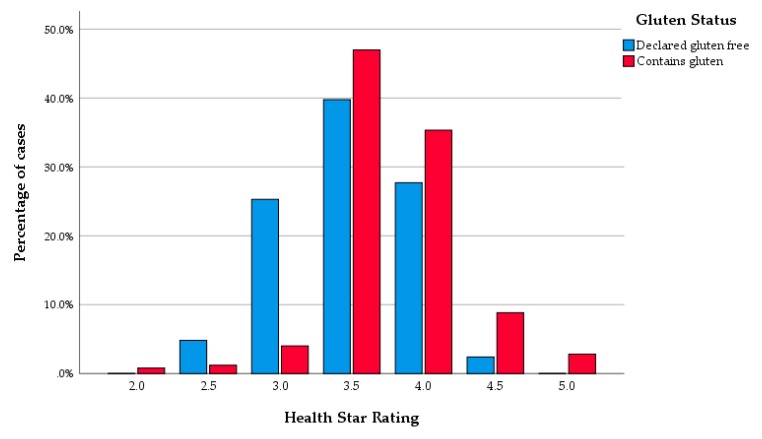
Health Star Rating of products according to gluten status.

A significantly lower protein content was observed in gluten-free products compared to products containing gluten. Otherwise there were no other significant differences in the nutritional qualities between gluten-containing products and declared gluten-free products. Accordingly, this resulted in a higher HSR of the gluten-containing products (Table 2 and Figure 2). The sensitivity analysis, where the products that lacked fibre content on the NIP were excluded, showed the same result; the HSR score was higher in gluten-containing products than in gluten-free products. A little over 5% of the supermarket’s own brand products were declared gluten-free; while about 33 % of the products were gluten-free among regular brands (Chi-Square *p* < 0.001). A significantly smaller proportion of the gluten-free bread products scored HSR ≥3.5 than the gluten-containing products (58 [70%] vs. 234 [94 %], *p* < 0.001).

## 4. Discussion

Our study of the healthiness of packaged bread products in Swedish supermarkets shows that the regular brands were healthier than supermarket’s own brands, though the difference was modest. The difference was primarily explained by the higher fibre content in regular brands. Due to its lower protein content, gluten-free bread was less nutritious than gluten-containing bread. Additionally, most of the bread products sold in Sweden are not labelled with the Keyhole symbol but, nevertheless, almost 90% of all bread was classified as healthy by the HSR system. 

The supermarket’s own brand products are increasing their market share in Sweden as well as in other countries [34,35]. Initially, the supermarket’s own brands were developed to provide lower prices, sometimes at the expense of quality, but the latest developments are shifting the focus more towards quality [34]. The retailers choose to have their own brands to ensure better quality control and to increase their financial margins. For the consumers, this development renders lower prices and more innovative products [34]. 

To our knowledge, only a few similar studies using HSR to investigate supermarket’s own brands have been done previously but with slightly different focus and methods compared to our study. These studies were conducted in Australia [35,48]. In alignment with our study, Kim et al. discovered that the HSR medians were the same but IQR were slightly higher in regular brands than among supermarket’s own brands in the bread category [48]. Similarly, the fibre content was found to be significantly higher in regular brands [48]. Unlike us, they found that supermarket’s own brands contained more saturated fat [48]. It is worth mentioning that Kim et al. excluded products that did not display the HSR on the packages which was not a criterion in our study; we included all the available products since there are no HSR labels on Swedish food products and we had access to the HSR calculation algorithm. In comparison, a study from the United States did not find differences in the main nutritional qualities between supermarket’s own brands and regular bread brands, although the sample size was small (*n* = 42) [49]. Additionally, there are likely differences in the food markets between different countries which makes it difficult to draw conclusions beyond the investigated market or country.

Another key finding in our study is that almost all of the bread products sold in the Swedish supermarkets are considered healthy according to HSR but only a small proportion carry the voluntary Keyhole symbol. It is very likely that many more bread products could be eligible for carrying the Keyhole symbol, but the manufacturers have chosen not to apply for it. The acquirement process of the Keyhole by the manufacturers and retailers might not be convenient enough and research to further evaluate this problem is currently in progress [50]. Currently, the information on Swedish packaged bread are not easily accessible in terms of health claims and likewise difficult for the consumer to evaluate the healthiness of the intended buy or compare it with other products. Even though the Keyhole symbol is undoubtedly important, due to it being well recognised by Swedish consumers and having strict labelling policies, Swedish consumers might benefit from additional nutrient profiling systems, such as the HSR, which aim to rank food products for easier comparison. The HSR system points out unhealthy products as well which has no equivalent in comparison to the Keyhole label. The downfault with both mentioned nutrient profiling systems are that if mandatory systems are not in place, the labelling will highlight healthy products to a greater extent. In our study, the HSR categorisation aligned with the Keyhole symbol in being specific for identifying healthy products but not sensitive. 

Comparing the products by gluten status showed that the nutritional qualities of declared gluten-free products were different compared to non-gluten free products on the market. Gluten-free bread products were less healthy than non-gluten free products due to lower protein content. Given that gluten is the protein part of the wheat grain (often the main component of bread), the result was expected and in alignment with previous studies [51,52,53]. An almost identical study with larger sample size conducted by Wu et al. in 2015 showed no significant difference for the HSR between gluten-free and gluten-containing bread in the Australian market [54]. However, they similarly found that the gluten-free bread products contain significantly less protein but not enough to influence the HSR [54]. In contrast, the study by Calvo-Lerma et al. noticed significantly higher total fat contents of the gluten-free bread products which is a finding that our study does not support [53]. However, this study was on the Spanish market and had a smaller sample size of bread compared to ours. 

The strength of our study is that we specifically aimed to include all the available packaged soft bread and not focus only on pre-labelled HSR products. Access to the algorithm and CMS database provided a standardised way to insert and analyse the nutritional qualities of the Swedish products which allows for analysis and comparison with other countries. That two researchers checked the inserted data into the database reduced the possibility for errors. We estimate that we collected a representative sample of all the packaged bread products sold in Sweden. During data collection, one visited store (Willy’s) refused to give access to their products. However, the store was part of the Axfood family and therefore, the store’s own branded available products were expected to be similar to those collected in Hemköp.

The limitations are that the results might not be generalizable to other countries, other food groups or separately sold non-packaged bread. Secondly, we excluded hard and dry bread products due to difficulties to categorise them properly according to the Australian HSR categorisation system used in the database. However, since hard and dry bread consumption is a lot lower than the soft bread consumption in Sweden [16], we do not find this to be a major issue with the study. Thirdly, the cross-sectional design is limited to a certain point in time while the food market and available products change constantly according to trends and other aspects [8]. 

A large proportion (11%) of the products did not display the fibre content. Unfortunately, the sample was too small to investigate the qualities of these products more deeply. Currently, it is not mandatory (unless a claim is made) in the EU (or Sweden) to display the fibre content even though the evidence suggests that low dietary fibre associates with life-threatening chronic diseases [1,2]. This lack of fibre content brought forth a minor issue in our study: the HSR formula was forced to use the average of fibre reported in the same category of products from the CMS database; but since the category definitions were not entirely consistent and applicable to Swedish breads these CMS-derived averages might have resulted in data distortion of the HSR. However, the sensitivity analyses excluding products without reported fibre content did not alter the main HSR outcomes.

Our study is the first study in Sweden to investigate the healthiness of products by applying HSR and it seems that this approach might be worth investigating, and even implementing, in the future. Even though the HSR has been developed in Australia, we have showed that the HSR aligns with the Nordic Keyhole symbol. The FoodSwitch platform aims to drive reformulation of products and empower consumers to buy healthier choices [31].

## 5. Conclusions

In conclusion, we found that the regular brand packaged bread products were overall slightly healthier than the supermarket’s own brands, which was driven by a higher fibre content in the regular brand products. Gluten-free bread products were slightly less healthy than gluten-containing bread due to lower protein content. 

## Figures and Tables

**Table 1:** Nutritional qualities of different bread products in Swedish supermarkets divided in supermarket own brand products and regular products.

Nutritional Qualities		All Products (*n* = 332)	Supermarket’s Own Brands (*n* = 95)	Regular Brands (*n* = 237)	*p*-Value ^1^
**Health Star Rating**	Mean (SD)	3.7 (0.5)	3.6 (0.5)	3.7 (0.5)	0.046
Energy (kcal) Per 100 g	Mean (SD)	263 (34)	263 (33)	263 (35)	0.58
Protein Per 100 g	Mean (SD)	7.6 (3.7)	8.3 (2.4)	7.3 (4.1)	0.009
Total Fat Per 100 g	Mean (SD)	4.1 (2.7)	3.8 (3.0)	4.2 (2.6)	0.021
Saturated Fat Per 100 g	Mean (SD)	0.7 (0.7)	0.8 (1.1)	0.6 (0.4)	0.23
Carbohydrate Per 100 g	Mean (SD)	46 (9.7)	47 (7.7)	46 (10)	0.23
Sugars Per 100 g	Mean (SD)	4.5 (3.1)	3.7 (2.9)	4.8 (3.2)	0.002
Salt Per 100 g	Mean (SD)	1.0 (0.3)	1.1 (0.3)	1.0 (0.3)	0.27
Fibre Per 100 g ^2^	Mean (SD)	5.1 (2.8)	4.3 (2.7)	5.4 (2.8)	<.001
Health Category ^3^	Healthy, *n* = 292		82 (86%)	210 (89%)	0.56
	Unhealthy, *n* = 40		13 (14%)	27 (11%)	

^1^ Mann–Whitney U-test, ^2^ Total of 35 products were missing information on the NIP for fibre content, ^3^ Chi-Square test.

**Table 2:** Nutritional qualities of different bread products in Swedish supermarkets divided by their gluten content.

Nutritional Qualities		Declared Gluten-Free	Contains Gluten	*p*-Value ^1^
**Health Star Rating**	Mean (SD)	3.5 (0.5)	3.8 (0.5)	<.001
Energy (kcal) Per 100 g	Mean (SD)	260 (41)	264 (32)	0.12
Protein Per 100 g	Mean (SD)	3.4 (2.0)	8.9 (3.1)	<.001
Total Fat Per 100 g	Mean (SD)	4.2 (2.9)	4.1 (2.6)	0.88
Saturated Fat Per 100 g	Mean (SD)	0.7 (0.4)	0.7 (0.7)	0.098
Carbohydrate Per 100 g	Mean (SD)	49 (12)	46 (8.6)	0.097
Sugars Per 100 g	Mean (SD)	4.6 (2.8)	4.4 (3.2)	0.17
Salt Per 100 g	Mean (SD)	1.0 (0.4)	1.0 (0.3)	0.45
Fibre Per 100 g	Mean (SD)	5.4 (2.8)	5.0 (2.9)	0.27

^1^ Mann-Whitney U-test.

## Supplementary Materials

The following are available online at https://www.mdpi.com/2072-6643/12/4/1162/s1, Table S1: The six different food categories in the Health Star Rating (HSR) system, Table S2: Health Star Rating (HSR) Baseline Points for Category 1, 1D, 2 or 2D Foods, Table S3: Health Star Rating (HSR) Protein, Fiber and Fruit/Vegetable/Nuts/Legumes Points, Table S4: Final scores used to assign Heath Star Ratings (HSR).
