# Supplementary material for: A Comparison of the Nutritional Qualities of Supermarket’s Own and Regular Brands of Bread in Sweden
Source: Nutrients. 2020 Apr 22;12(4):1162. doi: 10.3390/nu12041162 (PMC7230986; doi:10.3390/nu12041162)
Supplement: Supplementary file 1 [file nutrients-12-01162-s001.pdf]

Supplementary Table S1. The six different food categories in the Health Star Rating (HSR) system. Bread products are assigned to category 2.

Category 1	Beverages other than dairy beverages
Category 1D	Dairy beverages
Category 2	All foods other than those included in Category 1, 1D, 2D, 3 or 3D
Category 2D	Dairy foods other than those included in Category 1D or 3D
Category 3	Oils and spreads
Category 3D	Cheese and processed cheese (with calcium content >320mg/100g)

Supplementary Table S2. Health Star Rating (HSR) Baseline Points for Category 1, 1D, 2 or 2D Foods.

Baseline points	Average energy content (kJ) per 100 g or 100 mL	Average saturated fatty acids (g) per 100 g or 100 mL	Average total sugars (g) per 100 g or 100 mL	Average sodium (mg) per 100 g or 100 mL
0	≤335	≤1.0	≤5.0	≤90
1	>335	>1.0	>5.0	>90
2	>670	>2.0	>9.0	>180
3	>1005	>3.0	>13.5	>270
4	>1340	>4.0	>18.0	>360
5	>1675	>5.0	>22.5	>450
6	>2010	>6.0	>27.0	>540
7	>2345	>7.0	>31.0	>630
8	>2680	>8.0	>36.0	>720
9	>3015	>9.0	>40.0	>810
10	>3350	>10.0	>45.0	>900
11	>3685	>11.2	>49.0	>1005
12		>12.5	>54.0	>1121
13		>13.9	>58.0	>1251
14		>15.5	>63.0	>1397
15		>17.3	>67.0	>1559
16		>19.3	>72.0	>1740
17		>21.6	>76.0	>1942
18		>24.1	>81.0	>2168
19		>26.9	>85.0	>2420
20		>30.0	>90.0	>2701
21		>33.5	>94.0	>3015
22		>37.4	>99.0	>3365
23		>41.7		>3756
24		>46.6		>4192
25		>52.0		>4679
26		>58.0		>5223
27		>64.7		>5829
28		>72.3		>6506
29		>80.6		>7262
30		>90		>8106

The information is available at: http://healthstarrating.gov.au/internet/healthstarrating/publishing.nsf/Content/guide-for-industry-document.

Supplementary Table S3. Health Star Rating (HSR) Protein, Fiber and Fruit/Vegetable/Nuts/Legumes Points.

Points	Protein (g) per 100 g or 100 mL	Dietary fiber (g) per 100 g or 100 mL	% concentrated fruit or vegetables	% fruit vegetable nuts and legumes
0	≤1.6	≤0.9	<25	≤40
1	>1.6	>0.9	≥25	>40
2	≥3.2	>1.9	≥43	>60
3	>4.8	>2.8	≥52	>67
4	>6.4	>3.7	≥63	>75
5	>8.0	>4.7	≥67	>80
6	>9.6	>5.4	≥80	>90
7	>11.6	>6.3	≥90	>95
8	>13.9	>7.3	100	100
9	>16.7	>8.4		
10	>20.0	>9.7		
11	>24.0	>11.2		
12	>28.9	>13.0		
13	>34.7	>15.0		
14	>41.6	>17.3		
15	>50.0	>20.0		

The information is available at: http://healthstarrating.gov.au/internet/healthstarrating/publishing.nsf/Content/guide-for-industry-document.

Supplementary Table S4. Final scores used to assign Heath Star Ratings (HSR) depending on which food category the packaged product is assigned to. Packaged bread products belong to category 2.

**Health Star Rating**	**Food Category 1 Non-dairy beverage**	**Food Category 1D Dairy beverage**	**Food Category 2 Non-dairy foods**	**Food Category 2D Dairy foods**	**Food Category 3 Oils and spreads**	**Food Category 3D Cheese >320 mg Ca/100g**
5	≤ -6	≤ -2	≤ -11	≤ -2	≤ 13	≤ 22
4.5	-5	-1	-10 to -7	-1	14 to 16	23 to 24
4	-4	0	-6 to -2	0	17 to 20	25 to 26
3.5	-3	1	-1 to 2	1	21 to 23	27 to 28
3	-2	2	3 to 6	2	24 to 27	29 to 30
2.5	-1	3	7 to 11	3	28 to 30	31 to 32
2	0	4	12 to 15	4	31 to 34	33 to 34
1.5	1	5	16 to 20	5	35 to 37	35 to 36
1	2	6	21 to 24	6	38 to 41	37 to 38
0.5	≥3	≥7	≥25	≥7	≥42	≥39

The information is available at: http://healthstarrating.gov.au/internet/healthstarrating/publishing.nsf/Content/guide-for-industry-document.
